# Comparison between open reduction with internal fixation to circular external fixation for tibial plateau fractures: A systematic review and meta-analysis

**DOI:** 10.1371/journal.pone.0232911

**Published:** 2020-09-17

**Authors:** Zheng Li, Ping Wang, Li Li, Changshu Li, He Lu, Chuanshuang Ou

**Affiliations:** Department of Orthopaedics, Shenzhen Pingle Orthopaedic Hospital (Shenzhen Pingshan Traditional Chinese Medicine Hospital), Shenzhen, Guangdong, P.R. China; Assiut University Faculty of Medicine, EGYPT

## Abstract

Peer-reviewed published studies on tibial plateau fractures treated with either open reduction with internal fixation (ORIF) or circular external fixation were reviewed to compare functional, radiological outcomes, postoperative complications, and reoperation rates between the two methods. A systematic search of various databases including Medline, Cochrane Controlled Register of Trials (CENTRAL), ScienceDirect, and Google Scholar from inception until June 2019 was performed. 17 studies with 1168 participants were included in the review. Most of the studies (76%) were retrospective in nature and had low or unclear bias risks. Incidence of total infection (Odds ratio [OR], 2.58; 95% CI, 1.33–5.02) and malunions (OR, 2.56; 95% CI, 1.12–5.84) were higher and length of hospital stay was shorter in patients treated with circular external fixator (Mean difference [MD], -6.1; 95% CI, -11.1–-1.19). There were no differences in the incidence of secondary osteoarthritis (OR, 1.49; 95% CI, 0.92–2.42), range of motion (MD, 2.28; 95% CI, -11.27–15.82) non-union (OR, 1.44; 95% CI, 0.14–14.27) and reoperation rates (OR, 1.84; 95% CI, 0.90–3.78) between the two groups. Results from this investigation suggest that circular fixation may offer some advantages over ORIF such as a shortened length of hospital stay and early return to preinjury activities. Definitive clinical recommendations cannot be made as it also presents higher rates of postoperative complications than ORIF.

## Introduction

The incidence of tibial plateau fractures has increased over the past decade [1]. These fractures have a bimodal distribution affecting younger children (due to high energy traumas) and elderly individuals (due to increased bone fragility) [2]. Timely and adequate management of these injuries is important to avoid complications like knee instability or stiffness, secondary osteoarthritis (OA), compartment syndrome, and soft tissue damage [3]. Tibial plateau fractures can be managed either by conservative methods or by operative treatment. Anatomical reduction and restoration of alignment is important to prevent secondary displacement of the fracture fragments and is most commonly achieved by open reduction and internal fixation (ORIF) with plates and/or screws [4]. ORIF of tibial plateau fractures, however, is associated with significant complications like skin necrosis, early-onset osteoarthritis, and infection, especially in complex fractures and cases with significant soft tissue injury [5]. To overcome these drawbacks, minimally invasive methods have been developed which stabilize bone fragments by means of a circular external framework. External fixation usually requires a small and limited number of incisions thereby avoiding the detrimental effects of soft tissue dissection and preventing devascularisation of the osseous fragments [2, 6, 7].

Several studies have compared outcomes with the two treatment modalities for the management of tibial plateau fractures, but with conflicting results. A few systematic reviews and meta-analyses have also attempted to summarize existing evidence on the superiority of one treatment over the other [8–10]. Boutefnouchet et al [8] in their systematic review of 5 studies and Metcalfe et al [9] in their meta-analysis of 7 studies demonstrated similar results with either fixation technique for tibial plateau fractures. Later in 2017, in a meta-analysis of 11 studies, Zhao et al [10] also reported similar results but pointed out the deficiency of high-quality studies in the literature comparing the two fixation methods. Since then many new studies have been published and there is a need for a comprehensive and updated meta-analysis to provide quality evidence on this controversial topic. Therefore, the purpose of this study was to perform an updated literature search and meta-analysis comparing outcomes of ORIF with circular external fixation for the management of tibial plateau fractures.

## Material and methods

### Inclusion criteria and search strategy

A systematic search to identify studies that directly compared the effectiveness ORIF to circular external fixation for the management of tibial plateau fractures was performed. An electronic search was conducted from the following databases: Medline, ScienceDirect, Cochrane Bone, Joint and Muscle Trauma Group Specialised Register, Cochrane Central Register of Controlled Trials (CENTRAL), clinical trial registries like ClinicalTrials.gov, and WHO International Clinical Trials Registry Platform (ICTRP). The search performed used a combination of medical subject heading (MeSH) and free-text terms including "tibia", "tibial Fractures" "fractures", "open fracture reduction", "internal fixation", "circular fixator", "surgical fixation", "postoperative complications" and “wounds and injuries” for all English language publications from databases inception to June 2019. The search strategy of the MEDLINE database is presented as S1 File. The references list of all the primary trials obtained through the electronic search was also included and relevant articles were included in the analysis. Authors were contacted by email for missing data.

Inclusion criteria were all randomized or quasi-randomized controlled trials (RCT), and prospective or retrospective cohort studies comparing ORIF and circular external fixation for tibial plateau fracture management. Outcomes of the study were to include either radiographic outcome (secondary osteoarthritis [OA]), functional outcomes (range of motion, length of hospital stay) or postoperative complications (both superficial and deep infections), malunion, non-union, stiffness, thromboembolism and reoperation rate.

## Data extraction and outcomes

Two independent investigators performed the literature search. Articles were screened by their titles and abstracts for possible inclusion in the review. Full-text articles of relevant studies were obtained for final scrutiny based on inclusion criteria. Disagreements between investigators were resolved by consultation with another investigator. The Preferred Reporting Items for Systematic Review and Meta-Analysis (PRISMA) guidelines were followed for reporting this review [11]. The work has been reported in line with AMSTAR (Assessing the methodological quality of systematic reviews) Guidelines.

Two reviewers extracted the following details from the included studies:

1. General information: Date of extraction, study title, and authors
2. Methods: Study design, participants, and study setting
3. Participants: Total number of participants in each arm, baseline, and end line outcome measures, and inclusion and exclusion criteria.
4. Interventions: details of the intervention and comparison groups and follow-up duration
5. Outcomes: primary and secondary outcomes, time of outcome assessment, and other details for assessing the quality of studies

### Risk of bias

Two independent investigators assessed the risk of bias for included RCTs using the Cochrane risk of bias tool [12], focusing on the following domains: random sequence generation, allocation concealment, blinding of the participants and outcome assessment, incomplete outcome data, selective reporting of outcome and other sources of bias. For non-randomized studies, the risk of a bias assessment tool for non-randomized studies was used [13] based on the following domains: the selection of participants, confounding variables, intervention measurements, blinding of outcome assessment, incomplete outcome data, and selective outcome reporting.

### Statistical analysis

The software “Review Manager” (RevMan, version 5.3; Nordic Cochrane Centre [Cochrane Collaboration], Copenhagen, Denmark; 2014) was used for the meta-analysis. Continuous variables were summarized using Mean Difference with 95% confidence intervals (CI). Categorical variables were summarized using Mantel-Haenszel Odds Ratios (OR) with 95% CI. Predicting heterogeneity in the included studies, due to different study types and other methodological variations, we preferred a random-effects model to calculate the pooled effect size for all analyses [14]. Heterogeneity was classified according to I^2^ as mild (I^2^ <25%) moderate (I^2^ between 25 and 75%) or substantial (I^2^ >75%) [15]. Study-specific and pooled estimates were graphically depicted through forest plots. Publication biases for postoperative infection rates were assessed as other outcomes did not have the required number of studies to assess the publication bias (minimum of 10 studies) and this was graphically represented by a funnel plot.

## Results

**Fig 1:**
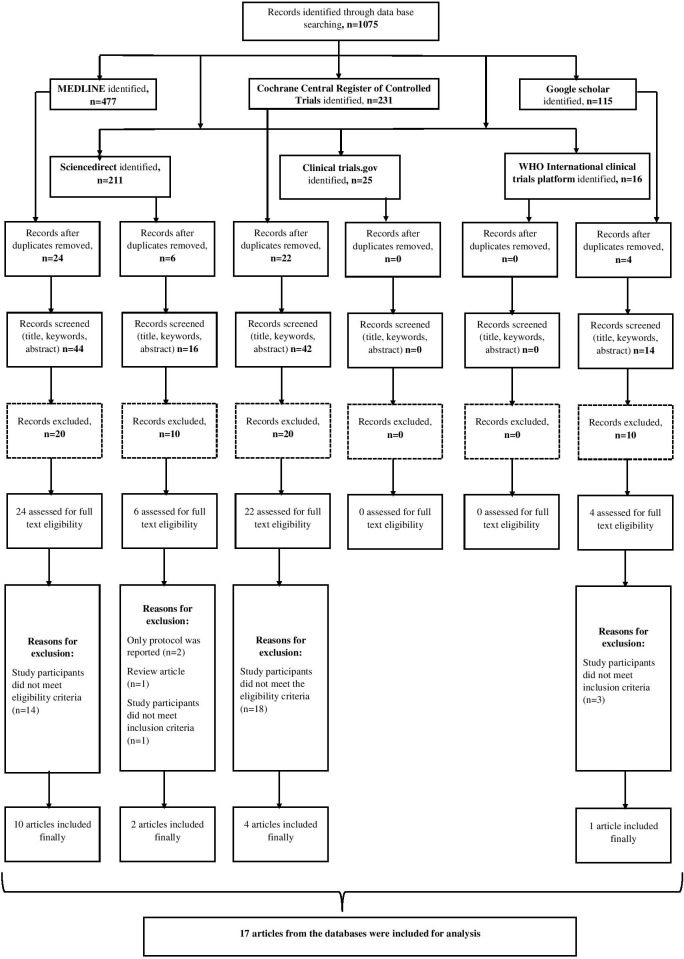
PRISMA flow chart showing the selection of studies for the current review.

Search flow chart of the study is presented in Fig 1. A total of 17 studies with 1168 participants were included in the meta-analysis.

Table 1 lists the characteristics of the studies analyzed. Two studies were RCTs, 2 were prospective, and 13 studies were retrospective. The mean age of study participants ranged from 12.4 to 55.7 years in the ORIF cohort, and that in the circular external fixation cohort ranged from 13.6 to 50.4 years. Of the 1168 participants, 550 patients were treated with the circular external fixator and 618 with ORIF. The sample sizes of studies in the ORIF cohort varied from 7 to 79 patients and in the circular external fixation arm from 2 to 85. Among the 17 studies included, 13 reported on postoperative infections (both superficial and deep infections), 10 reported reoperation rates, 6 reported radiographic evidence of secondary osteoarthritis, 5 reported on postoperative venous thromboembolism, malunion, and length of hospital stay, 4 reported on knee stiffness, and 3 reported cases of non-union and ranges of motion. Details of complications and reoperations in the included studies is presented in Table 2.

**Table 1:** Characteristics of the included studies.

Author and year	Country	Study Design	Sample size		Interventions	Follow up	Mean age (years)		Mean healing time	
			ORIF	External fixation			ORIF	External fixation	ORIF	External fixation
Ahearn 2014 [16]	United Kingdom	Retrospective	34	21	**External fixation:** Taylor Spatial Frame (TSF) **ORIF:** Incision: Unknown. Fixation: Lateral locking plate ± medial plate fixation	**External fixation:** mean 31 months (range 12–58 months) **ORIF:** mean 41 months (range 12–64 months)	NR separately (mean age of total participants = 44 years)		NR	
Bertrand 2017 [17]	Spain	Prospective	26	67	**External fixation:** TenXor Hybrid External Fixator **ORIF**: Incision: anteromedial and posterolateral; Fixation: Buttress plating	Follow up at 3, 6,18, and 24 months.	NR separately (mean age of total participants = 46.7 years)		19.28 weeks	22.83 weeks
Berven 2018 [18]	Denmark	Retrospective	68	62	**External fixation:** Ilizarov circular frame **ORIF**: Incision: Unknown Fixation: Locking plates	Unknown	50.44	55.74	NR	
Boston (Malik) 1994 [19]	Boston, USA	Retrospective	7	10	**External fixation:** Monticelli Spinellib circular fixator **ORIF:** Incision: Unknown. Fixation: Bilateral buttress, semi-tubular plates, or cannulated screws	**External fixation:** mean 10 months (range 5–28 months) **ORIF:** mean 33 months (range 6–60 months)	NR		NR	
Bove 2018 [20]	Italy	Retrospective	14	14	**External Fixation:** Taylor Spatial Frame (9/14); Ring Rod System (1/14); Truelok Hexapod System (4/14); **ORIF**: Incision: Unknown; Fixation: fixed angle locking plating using the Less Invasive Stabilization System	Follow-up evaluation was continued either until radiographic Healing or up to a year after surgery.	43	51	17 weeks	22 weeks
Chan 2012 [21]	United Kingdom	Retrospective	24	35	**External fixation:** Ilizarov circular frame (23/35, 65.7%), Hoffman IIb with limited internal fixation (13/35, 37.1%) **ORIF:** Incision: Unknown. Fixation: Buttress plate (21/24, 84%), Less Invasive Stabilization Systemic (4/24, 16%)	3, 6, 12, and 24 months post-injury	45.04	52.03	NR	
Chertsey (Nawaz) 2013 [22]	United Kingdom	Retrospective	79	45	**External fixation:** Ilizarov circular frame **ORIF:** Incision: Unknown. Fixation: Unknown	Unknown	NR		NR	
Conserva 2015 [23]	Italy	Retrospective	38	41	**External Fixation:** Circular external fixator frame **ORIF**: Incision: combined medial and lateral incision was used for Schatzker type V and VI, For type IV fractures, a the direct posteromedial approach was used; Fixation: temporary fixation with Kirschner wires or interfragmentary screw	Mean follow-up was 39.4 months (13–72) for the External Fixation group and 35.1 months (12–68) for ORIF group	NR separately (mean age of total participants = 54.1 years)		17.2 weeks	15.9 weeks
COTS 2006 [24]	Canada	Randomized Controlled Trial	40	43	**External fixation:** Closed/percutaneous/limited reduction, percutaneous lag screw, and Ilizarov circular frame **ORIF:** Incision: Single anterior or combined medial/lateral. Fixation: medial and lateral non-locking buttress plates ± iliac crest bone grafting	6, 12, and 24 months post-injury	43.3	46.2	NR	
Guryel 2010 [25]	United Kingdom	Retrospective	79	45	**External fixation:** Ilizarov circular frame **ORIF:** Incision: Unknown. Fixation: Unknown	Unknown	NR		NR	
Hao 2019 [26]	China	Retrospective	67	85	**External Fixation:** Frames of the Hoffmann II External Fixation **Limited Internal Fixation**: Incision: Unknown Fixation: including cortical screws and the Ni-Ti arched shape-memory connector	Mean follow-up time 17.15 months (range: 12.00 to 24.00 months) in the External Fixation group and 16.20 months (range 12.00 to 19.00 months) in the Limited Internal Fixation group	42.17	45.31	5.84 months	6.19 months
Jansen 2013 [27]	Germany	Retrospective	20	2	**External fixation:** Synthes AOC fixator or Ilizarov circular frame **ORIF:** Incision: Unknown. Fixation: Less Invasive Stabilization System (LISS) (19/20, 95.0%) ± additional plates (7/20, 30.4%) ± artificial bone substitute (7/20,30.4%)	Mean 67 months (range 36–109 months)	NR separately (mean age of total participants = 46 years)		NR	
Kartheek 2017 [28]	India	Prospective	15	15	**External fixation:** Ilizarov circular frame **ORIF**: Incision: Unknown: LISS with standard locking plates and cancellous screws.	The mean follow-up period was 56.4 weeks.	44.73	43.20	NR	
Krupp 2009 [29]	USA	Retrospective	28	30	**External fixation:** Hoffman II Hybrid or circular frames and interval ORIF (locking plate or LISSc) **ORIF:** Incision: Unknown. Fixation: locking plate LISSc	Mean unknown (range 6–53 months)	NR		6 months	7 months
Lin 2018 [30]	China	Retrospective	37	13	**External fixation:** Circular External Fixator **ORIF:** Incision: Unknown. Fixation: compression or bridge plate fixation	The mean follow-up time was 15.8 months.	13.6	12.4	12.1 weeks	18.3 weeks
Pirani 2018 [31]	Canada	Randomized Controlled Trial	33	10	**External fixation:** Closed/percutaneous/limited reduction, percutaneous lag screw, and Ilizarov circular frame **ORIF:** Incision: Single anterior or combined medial/lateral. Fixation: medial and lateral non-locking buttress plates ± iliac crest bone grafting	6, 12, and 24 months post-injury	NR		NR	
Pun 2014 [32]	India	Retrospective	9	12	**External fixation:** Circular frame + medial percutaneous screws = 12 **ORIF**: dual plate = 9	Outcomes at 1 year Mean follow up duration 29 months	NR separately (mean age of total participants = 43.8 years)		NR	

ORIF, Open reduction internal fixation; NR, Not reported

**Table 2:** Details of complications and reoperations in included studies.

Study	Complications		Reoperations	
	ORIF	External fixation	ORIF	External fixation
Ahearn 2014 [16]	Deep peroneal nerve palsy (1) Screws backed out (2) Infection (2) Significant joint collapse (1)	Infections (6) Common peroneal nerve injury (1) Deep peroneal nerve palsy (1) DVT (1)	Implant removal (2) Bone grafting (1) Converted to external fixator (1)	-
Bertrand 2017 [17]	Infection (3) Consolidation delay (2) Malunion (5)	Infection (22) Consolidation delay (10) Malunion (16)	Total (5) Mobilization under anesthesia (1) Arthroscopic arthrolysis (1) Cannulated screw removal (2) Surgical debridement (3)	Total (22) Autograft (5) Mobilization under anesthesia (3) Arthroscopic arthrolysis (3) Surgical debridement (8) Fasciotomy (1)
Berven 2018 [18]	Non-union (9) Knee stiffness (9) Knee instability (3) Infection (7) Heterotopic ossification (9) Osteoarthritis (34) DVT (1) Malunion (15)	Non-union (3) Knee stiffness (7) Knee instability (5) Infection (31) Heterotopic ossification (1) Osteoarthritis (42) Peroneus paresis (3) DVT (2) Malunion (16)	NR	
Boston (Malik) 1994 [19]	Infection (3)	Infection (5)	NR	
Bove 2018 [20]	Non-union (1) peroneal nerve palsy (1)	Non-union (1)	NR	
Chan 2012 [21]	Infection (3) DVT (2) Compartment syndrome (3) Osteoarthritis (7)	Infection (9) DVT (4) Compartment syndrome (2) Osteoarthritis (9)	Fasciotomy (3) Wound debridement (2)	Fasciotomy (2) Wound debridement (2)
Chertsey (Nawaz) 2013 [22]	Infection (2) DVT (2)	-	NR	
Conserva 2015 [23]	Infection (6) Osteoarthritis (4)	Infection (5) Knee stiffness (3) Osteoarthritis (11)	NR	
COTS 2006 [24]	Osteoarthritis (11)	Osteoarthritis (13)	Split thickness skin graft (5)* Implant removal (8) Knee manipulation (3) Knee arthroplasty (2) Above knee amputation (1) Flap surgery (4) Revision ORIF (4)	Split thickness skin graft (2)* Implant removal (6) Knee manipulation (2) Knee arthroplasty (1)
Guryel 2010 [25]	DVT (2) Osteoarthritis (3)	DVT (4) Osteoarthritis (1)	Total knee replacement (3)	Total knee replacement (1)
Hao 2019 [26]	Infection (30) Non-union (1) Malunion (2)	Infection (36) Non-union (12) Malunion (15)	Change of fixation system (4)	Change of fixation system (29)
Jansen 2013 [27]	Infection (2) Compartment syndrome (1) Soft tissue necrosis (1) Pseudarthrosis (4)	Infection (2)	NR	
Kartheek 2017 [28]	Infection (1) Knee stiffness (4) Osteoarthritis (5) Knee stability (2) Minor hardware impingement (3) Varus collapse (3)	Infection (3) Knee stiffness (1) Osteoarthritis (3) Minor hardware impingement (1)	NR	
Krupp 2009 [29]	Infection (2) Knee stiffness (1) Painful hardware (2) Heterotopic bone formation (2) Delayed union (7) Knee instability (1) Malunion (2)	Infection (4) Knee stiffness (4) Painful hardware (3) Heterotopic bone formation (1) Delayed union (11) Knee instability (1) Gastrosoleus equinus (1) Malunion (12)	Debridement/Hardware removal (1)* Manipulation/change of fixation (2) Bone grafting (1) Scope & release/Meniscectomy (1) Hardware removal (2) Total knee arthroplasty (1)	Debridement/Hardware removal (95)* Manipulation/change of fixation (3) Bone grafting (3) Scope & release/Meniscectomy (3) Hardware removal (4) Total knee arthroplasty (1) Achilles tendon lengthening (1) Change of plate (2) Intramedullary nailing (1) Screw to tibial plateau gap (1)
Lin 2018 [30]	Malunion (2)	Loss of reduction (4) Infection (1) Limb length discrepancy (2) Malunion (2)	Reoperation due to malunion (2)	Reoperation due to loss of reduction (4) And malunion (2)
Pirani 2018 [31]	NR		Skin graft (3)* Quadricepsplasty (1) Manipulation (2) Muscle flap (2) Above knee amputation (1) Revision fixation (5) Bone graft (2) Bead pouch (3) Synovectomy (1) Sequestrectomy (1)	Manipulation (4) Revision fixation (2) Bone graft (1)
Pun 2014 [32]	-	Infection (2)	NR	

NR, not reported

Figure in parenthesis indicate number of patients with the complications unless specified otherwise

*Figure indicates total number of procedures

Assessments of risk of bias for RCTs and non-randomized studies is presented in Table 3. The percent of cases lost to follow-up in the included studies ranged from 0% to 12.5%. Both RCTs [24, 31] included in the review had low risks in almost all the domains except for high risk in the blinding of participants and outcome assessments, and they had an unclear risk of selective reporting of outcomes given that the published protocols for the studies could not be identified. Among the non-randomized studies, all the studies had a low risk of intervention measurements and unclear risks of selective reporting of outcomes (protocols not published) or blinding of outcome assessment (not mentioned in the studies). Almost all the studies except Jansen 2013 [27] had low risks with respect to the selection of participants. Four out of 15 had high risks of bias concerning incomplete outcome data due to loss to follow up of the study participants.

**Table 3:** Risk of bias assessment.

**A. Randomized studies, N = 2**							
**S. No**	**Study**	**Random sequence generation**	**Allocation concealment**	**Blinding of the participants, outcome assessment**	**Incomplete outcome data**	**Selective reporting of outcome**	**Other risk of bias**
1.	COTS 2006	Low risk	Low risk	High risk	Low risk	Unclear risk	Low risk
2.	Pirani 2018	Low risk	Low risk	High risk	Low risk	Unclear risk	Low risk
**B. Non-randomized studies, N = 15**							
**S. No**	**Study**	**Selection of participants**	**Confounding variable**	**Intervention measurement**	**Blinding of the outcome assessment**	**Incomplete outcome data**	**Selective reporting of outcome**
1.	Ahearn 2014	Low risk	High risk	Low risk	Unclear risk	High risk	Unclear risk
2.	Bertrand 2017	Low risk	High risk	Low risk	Unclear risk	Low risk	Unclear risk
3.	Berven 2018	Low risk	High risk	Low risk	Unclear risk	High risk	Unclear risk
4.	Boston (Malik) 1994	Low risk	High risk	Low risk	Unclear risk	High risk	Unclear risk
5.	Bove 2018	Low risk	High risk	Low risk	Unclear risk	Low risk	Unclear risk
6.	Chan 2012	Low risk	High risk	Low risk	Unclear risk	High risk	Unclear risk
7.	Chertsey (Nawaz) 2017	Low risk	High risk	Low risk	Unclear risk	Low risk	Unclear risk
8.	Conserva 2015	Low risk	High risk	Low risk	Unclear risk	Low risk	Unclear risk
9.	Guryel 2010	Low risk	High risk	Low risk	Unclear risk	Low risk	Unclear risk
10.	Hao 2019	Low risk	High risk	Low risk	Unclear risk	Low risk	Unclear risk
11.	Jansen 2013	Unclear risk	High risk	Low risk	Unclear risk	Low risk	Unclear risk
12.	Karthik 2017	Low risk	Low risk	Low risk	Unclear risk	Low risk	Unclear risk
13	Krupp 2009	Low risk	High risk	Low risk	Unclear risk	High risk	Unclear risk
14.	Lin 2018	Low risk	High risk	Low risk	Unclear risk	Low risk	Unclear risk
15.	Pun TB 2014	Low risk	High risk	Low risk	Unclear risk	Low risk	Unclear risk

**Fig 2:**
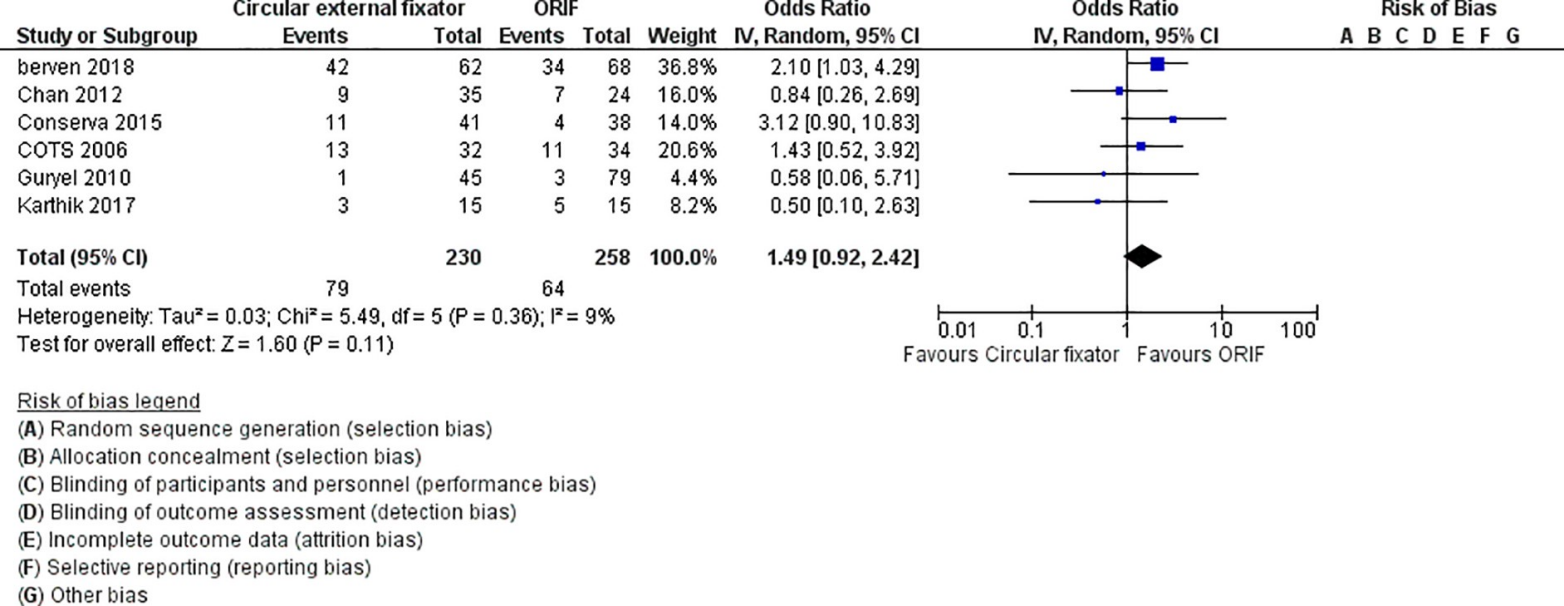
Forest plot for secondary osteoarthritis between ORIF and circular external fixator.

Among the included studies, 6 reported radiographic evidence of post intervention secondary OA. Meta-analysis indicated no significant difference between in the incidence of secondary OA between ORIF and circular external fixator groups (OR,1.49; 95%CI, 0.92–2.42; I^2^ = 9%; p = 0.11) (Fig 2).

**Fig 3:**
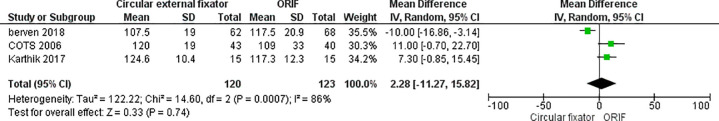
Forest plot for range of motion between ORIF and circular external fixator.

Three studies [18, 24, 28] reported data on post-operative knee ranges of motion in degrees. The pooled mean difference did not demonstrate any significant difference in range of motion between the two treatment modalities (MD,2.28; 95% CI, -11.27 to 15.82; I^2^ = 86%, p = 0.74) (Fig 3).

**Fig 4:**
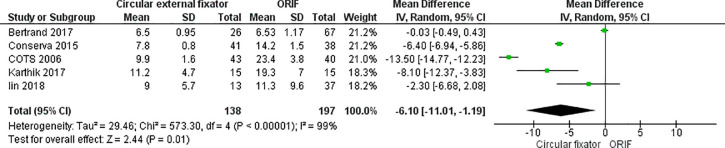
Forest plot for length of hospital stay between ORIF and circular external fixator.

Five studies [17, 23, 24, 28, 30] reported the length of hospital stay for both study cohorts. All the studies found the patients in the circular external fixation cohort had shorter hospital stays than those in the ORIF cohort (Fig 4). Our meta-analysis also indicated a significantly shorter hospital stay for patients treated with circular external fixator (MD, -6.10; 95% CI, -11.01 to -1.19; I^2^ = 99%, p = 0.01) (Fig 4)

**Fig 5:**
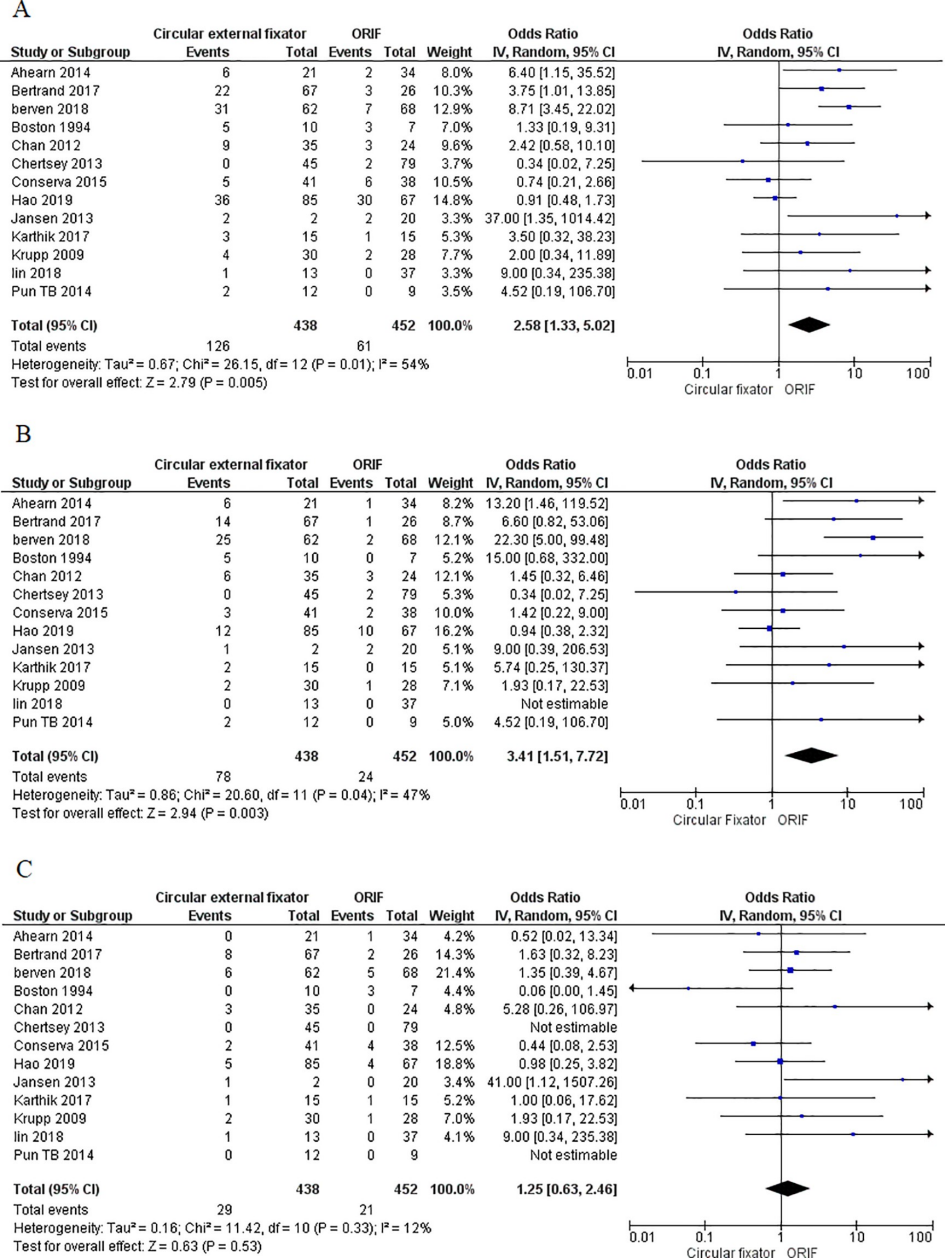
Forest plot for postoperative infection rate between ORIF and circular external fixator arm A. Total infections, B. Superficial infections, C. Deep infections.

In total, 13 studies reported the postoperative infection rates from both cohorts. Most studies, except for 3 (Chertsey 2013 [22], Conserva 2015 [23] and Hao 2019 [26], found the patients in the circular fixation arm were more likely to develop postoperative infections than the patients in the ORIF cohort. Meta-analysis indicated higher odds of total infections with ORIF as compared to circular external fixator (OR, 2.58; 95%CI, 1.33–5.02; I^2^ = 54%; p = 0.005) (Fig 5A). Data of superficial and deep infections were also pooled separately. Our results indicate significantly higher superficial infection rates with ORIF (OR, 3.41; 95%CI, 1.51–7.72; I^2^ = 47%; p = 0.003) (Fig 5B) but no difference in the incidence of deep infections between the two groups (OR, 1.25; 95%CI, 0.63–2.46; I^2^ = 12%; p = 0.53) (Fig 5C). No gross asymmetry was noted on funnel plot indicating the absence of publication biases (S2 and S3 Files).

**Fig 6:**
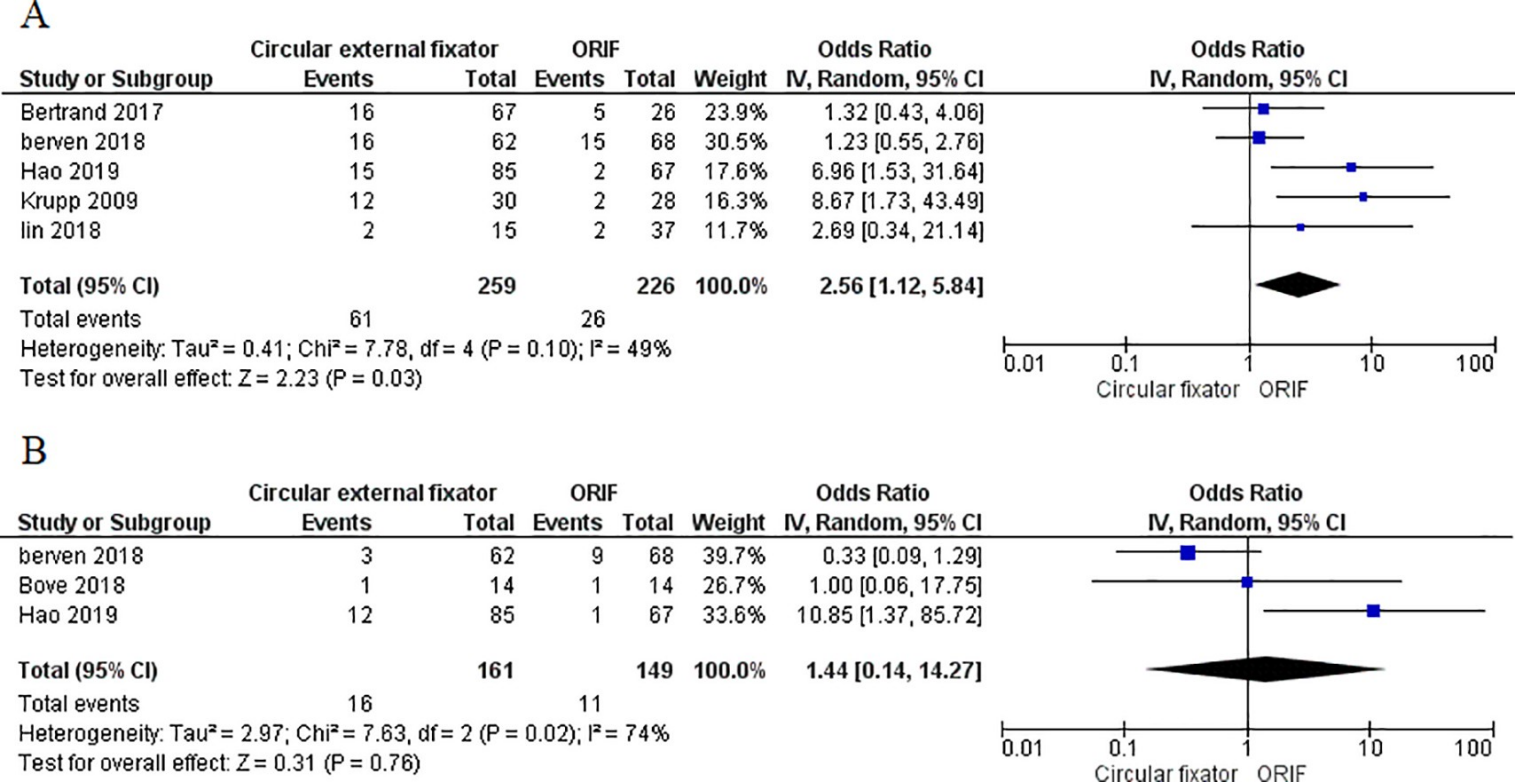
Forest plot for malunion and non-union between ORIF and circular external fixator A. Malunion, B. Non-union.

Five studies [17, 18, 26, 29, 30] reported data on malunions. Our meta-analysis indicates circular external fixator compared to ORIF results in a significantly higher chance of developing malunion (OR, 2.56; 95%CI, 1.12–5.84; I^2^ = 49%; p = 0.03) (Fig 6A). Three studies [18, 20, 26] reported the incidence of non-unions in the two groups. Pooled analysis indicates no difference in the incidence of non-union with either methods of management (OR, 1.44; 95%CI, 0.14–14.27; I^2^ = 74%; p = 0.76) (Fig 6B)

**Fig 7:**
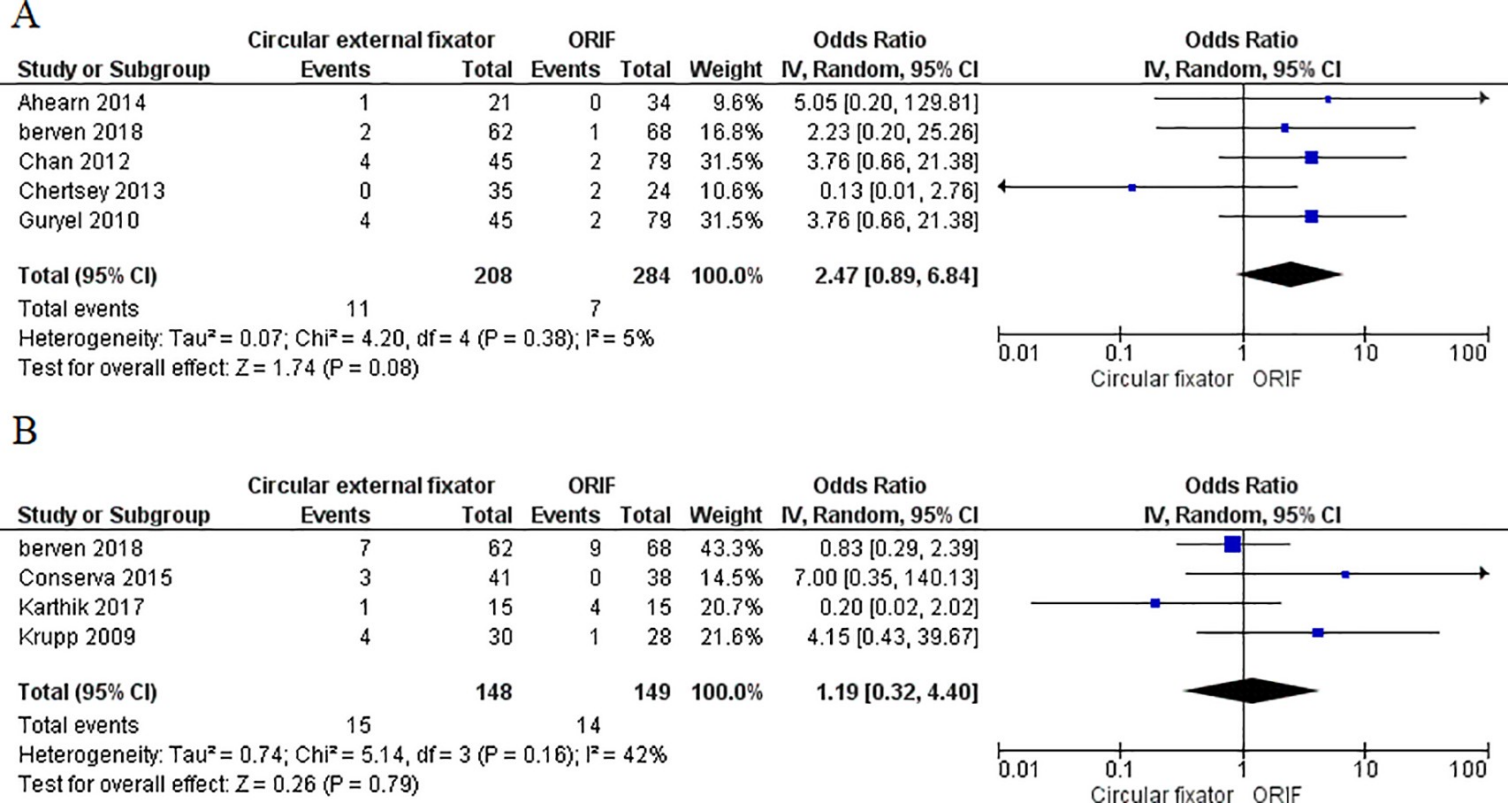
Forest plot for thromboembolism and knee stiffness between ORIF and circular external fixator A. Thromboembolism B. Knee stiffness.

**Fig 8:**
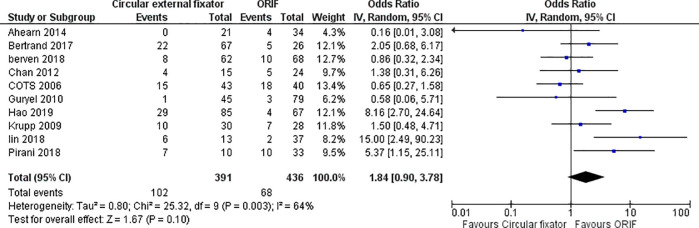
Forest plot showing the difference in reoperation rates between ORIF and circular external fixator arm (n = 10).

Five studies [16, 18, 21, 22, 25] reported the incidence of postoperative thromboembolism and four studies reported data on knee stiffness [18, 23, 28, 29] in both study groups. Meta-analysis demonstrated no difference in the incidence of postoperative thromboembolism (OR, 2.47; 95%CI, 0.89–6.84; I^2^ = 10%; p = 0.08) (Fig 7A) and knee stiffness (OR, 1.19; 95%CI, 0.32–4.40; I^2^ = 42%; p = 0.79) (Fig 7B) between ORIF and circular external fixator. Ten studies reported reoperation rates from both study cohorts. Pooled results did not demonstrate any difference in reoperation rates with ORIF or circular external fixator (OR, 1.84; 95%CI, 0.90–3.78; I^2^ = 64%; p = 0.10) (Fig 8).

## Discussion

The management of tibial plateau fractures has seen technological advances [2, 6, 7]. A lack of systematic and high-quality research assessing the effectiveness of these newer techniques make it difficult to determine if the safety and efficacy surpass previous standards of care. The purpose of this analysis was to compare the efficacies of ORIF to circular external fixation, in terms of functional and radiological outcomes, postoperative complications, and reoperation rates among patients with tibia plateau fracture. A total of 17 studies consisting of 1168 participants met the inclusion criteria for this analysis. Out of these, only 2 studies were RCTs and 2 were prospective studies, while the remainder (76% of the studies) were retrospective studies. Most of the studies in this review had either unclear or low bias risks. Substantial heterogeneity among the reported outcomes in the studies was observed. Given the small number of studies under each of the outcomes (less than 10) a meta-regression to explore the source of heterogeneity could not be performed. Except for the length of hospital stay, all other outcomes (functional and radiological outcomes, and postoperative complication and reoperation rates) were superior after ORIF than after circular external fixation. However, the only conclusive and significant evidence for the risk of postoperative complications such as all infections, superficial infections, and malunions were more frequent in patients undergoing external circular fixation. The pooling of all other variables amongst studies did not show any statistically significant differences in terms of functionality, radiological outcomes, and complications between the study cohorts.

Patients undergoing circular external fixations were more likely to develop postoperative infections and malunions than the patients undergoing ORIF. However, patients who undergo circular external fixations return to their preinjury activities sooner than patients who underwent ORIF surgery. Similar findings were reported in the review by Metcalfe et al (2015) [9] in which authors found conclusive evidence for postoperative infections (all infections and superficial infections) being more frequent in patients after external fixations than after ORIF. That review included only 7 studies and functional outcomes (range of motion, length of hospital stay) and postoperative complications (knee stiffness, malunions, and non-union) were not assessed. Results from this investigation provide evidence to aid surgeons to determine which surgical intervention is preferable for their patients with tibia plateau fractures.

The major strengths of this study include the comprehensive literature search and the broad search strategy to capture relevant publications. Besides, this review compares functional outcomes such as knee range of motion and postoperative complications (malunions, non-unions, and knee stiffnesses) between patients treated by ORIFs or circular external fixations.

There were limitations in this analysis; there was a high level of heterogeneity across the studies included in the review and it was not possible to identify the source of heterogeneity as each of the outcomes was present in only a few studies (less than 10) making meta-regressions not possible. As a result, it was necessary to apply a random-effects model to account for this heterogeneity. Also, this review included only 2 RCTs among the 17 studies included. Since most of these studies were retrospective in nature, it is not possible to infer causal associations between the interventions and the outcomes. More trials of adequate size need to be conducted to make stronger recommendations on treatment. It was not possible to determine which fixation method between ORIF or circular external fixation is superior for the management of tibia plateau fractures. However, most of the studies in this investigation noted that the external fixator method was inferior to ORIF due to postoperative complication rates. Uncertainties regarding the functional and radiological outcomes persist. Adequately powered RCTs or prospective studies are needed to strengthen the evidence for recommendations on how to best manage patients with tibia plateau fractures.

## Conclusions

Circular fixator method may offer some advantages over ORIF such as shortened length of hospital stays and early returns to preinjury activities, however, had a higher rate of postoperative complications than the ORIF cohort. Inconclusive evidence in terms of the functional and radiological outcomes was observed. Future studies with larger sample sizes are needed to identify the best management strategy for tibia plateau fractures.

## Supporting information

S1 Checklist PRISMA 2009 checklist. (DOC)

S1 File Search strategy of MEDLINE database. (DOCX)

S2 File Funnel plot checking the publication bias among studies reporting postoperative infections (n = 13). (TIF)

S3 File Funnel plot checking the publication bias among studies reporting reoperation rates (n = 10). (TIF)
