# Supplementary material for: Comparison between open reduction with internal fixation to circular external fixation for tibial plateau fractures: A systematic review and meta-analysis
Source: PLoS One. 2020 Sep 17;15(9):e0232911. doi: 10.1371/journal.pone.0232911 (PMC7498044; doi:10.1371/journal.pone.0232911)
Supplement: S1 File — (DOCX) [file pone.0232911.s002.docx]

Supplementary file 1. Search terms and search strategy of MEDLINE.

| **Query** | **Search Details** |
| --- | --- |
| ((tibial Fractures) AND (circular fixator)) AND (wounds and injuries) | ((("tibial fractures"[MeSH Terms] OR ("tibial"[All Fields] AND "fractures"[All Fields])) OR "tibial fractures"[All Fields]) AND ((((((("circular"[All Fields] OR "circularization"[All Fields]) OR "circularize"[All Fields]) OR "circularized"[All Fields]) OR "circularizes"[All Fields]) OR "circularizing"[All Fields]) OR "circulars"[All Fields]) AND ((((((((("fixate"[All Fields] OR "fixated"[All Fields]) OR "fixates"[All Fields]) OR "fixating"[All Fields]) OR "fixation"[All Fields]) OR "fixational"[All Fields]) OR "fixations"[All Fields]) OR "fixator"[All Fields]) OR "fixator s"[All Fields]) OR "fixators"[All Fields]))) AND (("wounds and injuries"[MeSH Terms] OR ("wounds"[All Fields] AND "injuries"[All Fields])) OR "wounds and injuries"[All Fields]) |
| (((tibia) AND (fractures)) AND (surgical fixation)) AND (postoperative complications) | (((((("tibia"[MeSH Terms] OR "tibia"[All Fields]) OR "tibias"[All Fields]) OR "tibia s"[All Fields]) OR "tibiae"[All Fields]) AND ((((((((("fractur"[All Fields] OR "fractural"[All Fields]) OR "fracture s"[All Fields]) OR "fractures, bone"[MeSH Terms]) OR ("fractures"[All Fields] AND "bone"[All Fields])) OR "bone fractures"[All Fields]) OR "fracture"[All Fields]) OR "fractured"[All Fields]) OR "fractures"[All Fields]) OR "fracturing"[All Fields])) AND (((((("surgical procedures, operative"[MeSH Terms] OR (("surgical"[All Fields] AND "procedures"[All Fields]) AND "operative"[All Fields])) OR "operative surgical procedures"[All Fields]) OR "surgical"[All Fields]) OR "surgically"[All Fields]) OR "surgicals"[All Fields]) AND ((((((((("fixate"[All Fields] OR "fixated"[All Fields]) OR "fixates"[All Fields]) OR "fixating"[All Fields]) OR "fixation"[All Fields]) OR "fixational"[All Fields]) OR "fixations"[All Fields]) OR "fixator"[All Fields]) OR "fixator s"[All Fields]) OR "fixators"[All Fields]))) AND (("postoperative complications"[MeSH Terms] OR ("postoperative"[All Fields] AND "complications"[All Fields])) OR "postoperative complications"[All Fields]) |
| ((tibial Fractures) AND (internal fixation)) AND (circular fixator) | ((("tibial fractures"[MeSH Terms] OR ("tibial"[All Fields] AND "fractures"[All Fields])) OR "tibial fractures"[All Fields]) AND (((("fracture fixation, internal"[MeSH Terms] OR (("fracture"[All Fields] AND "fixation"[All Fields]) AND "internal"[All Fields])) OR "internal fracture fixation"[All Fields]) OR ("internal"[All Fields] AND "fixation"[All Fields])) OR "internal fixation"[All Fields])) AND ((((((("circular"[All Fields] OR "circularization"[All Fields]) OR "circularize"[All Fields]) OR "circularized"[All Fields]) OR "circularizes"[All Fields]) OR "circularizing"[All Fields]) OR "circulars"[All Fields]) AND ((((((((("fixate"[All Fields] OR "fixated"[All Fields]) OR "fixates"[All Fields]) OR "fixating"[All Fields]) OR "fixation"[All Fields]) OR "fixational"[All Fields]) OR "fixations"[All Fields]) OR "fixator"[All Fields]) OR "fixator s"[All Fields]) OR "fixators"[All Fields])) |
| (tibia) AND (open fracture reduction) | (((("tibia"[MeSH Terms] OR "tibia"[All Fields]) OR "tibias"[All Fields]) OR "tibia s"[All Fields]) OR "tibiae"[All Fields]) AND (("open fracture reduction"[MeSH Terms] OR (("open"[All Fields] AND "fracture"[All Fields]) AND "reduction"[All Fields])) OR "open fracture reduction"[All Fields]) |
| ((tibia) AND (fractures)) AND (circular fixator) | ((((("tibia"[MeSH Terms] OR "tibia"[All Fields]) OR "tibias"[All Fields]) OR "tibia s"[All Fields]) OR "tibiae"[All Fields]) AND ((((((((("fractur"[All Fields] OR "fractural"[All Fields]) OR "fracture s"[All Fields]) OR "fractures, bone"[MeSH Terms]) OR ("fractures"[All Fields] AND "bone"[All Fields])) OR "bone fractures"[All Fields]) OR "fracture"[All Fields]) OR "fractured"[All Fields]) OR "fractures"[All Fields]) OR "fracturing"[All Fields])) AND ((((((("circular"[All Fields] OR "circularization"[All Fields]) OR "circularize"[All Fields]) OR "circularized"[All Fields]) OR "circularizes"[All Fields]) OR "circularizing"[All Fields]) OR "circulars"[All Fields]) AND ((((((((("fixate"[All Fields] OR "fixated"[All Fields]) OR "fixates"[All Fields]) OR "fixating"[All Fields]) OR "fixation"[All Fields]) OR "fixational"[All Fields]) OR "fixations"[All Fields]) OR "fixator"[All Fields]) OR "fixator s"[All Fields]) OR "fixators"[All Fields])) |
| ((tibial Fractures) AND (open fracture reduction)) AND (circular fixator) | ((("tibial fractures"[MeSH Terms] OR ("tibial"[All Fields] AND "fractures"[All Fields])) OR "tibial fractures"[All Fields]) AND (("open fracture reduction"[MeSH Terms] OR (("open"[All Fields] AND "fracture"[All Fields]) AND "reduction"[All Fields])) OR "open fracture reduction"[All Fields])) AND ((((((("circular"[All Fields] OR "circularization"[All Fields]) OR "circularize"[All Fields]) OR "circularized"[All Fields]) OR "circularizes"[All Fields]) OR "circularizing"[All Fields]) OR "circulars"[All Fields]) AND ((((((((("fixate"[All Fields] OR "fixated"[All Fields]) OR "fixates"[All Fields]) OR "fixating"[All Fields]) OR "fixation"[All Fields]) OR "fixational"[All Fields]) OR "fixations"[All Fields]) OR "fixator"[All Fields]) OR "fixator s"[All Fields]) OR "fixators"[All Fields])) |
