# Supplementary figures and images for: Comparison between open reduction with internal fixation to circular external fixation for tibial plateau fractures: A systematic review and meta-analysis
Source: PLoS One. 2020 Sep 17;15(9):e0232911. doi: 10.1371/journal.pone.0232911 (PMC7498044; doi:10.1371/journal.pone.0232911)

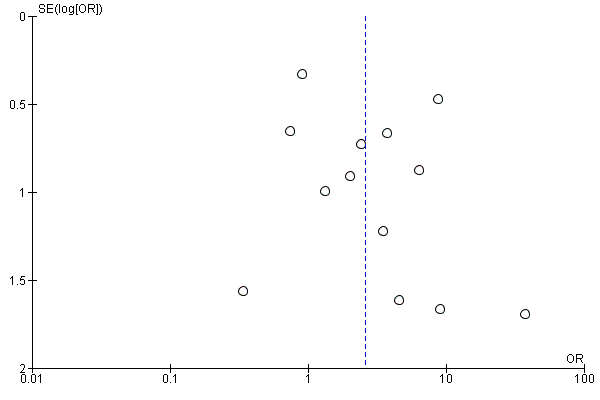

Supplement: S2 File — (TIF) [file pone.0232911.s003.tif]

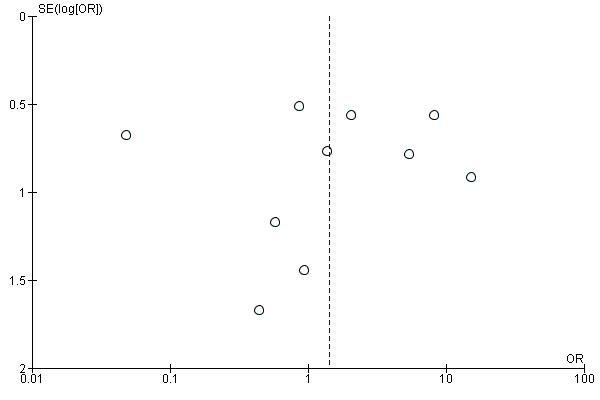

Supplement: S3 File — (TIF) [file pone.0232911.s004.tif]
